# Emerging genetics of COPD

**DOI:** 10.1002/emmm.201100627

**Published:** 2012-10-23

**Authors:** Annerose Berndt, Adriana S Leme, Steven D Shapiro

**Affiliations:** Division of Pulmonary, Allergy and Critical Care Medicine, Department of Medicine, University of Pittsburgh Medical Center, University of Pittsburgh School of MedicinePittsburgh, PA, USA

**Keywords:** COPD, genes, genetics, genome-wide association studies, obstructive pulmonary disease

## Abstract

Since the discovery of alpha-1 antitrypsin in the early 1960s, several new genes have been suggested to play a role in chronic obstructive pulmonary disease (COPD) pathogenesis. Yet, in spite of those advances, much about the genetic basis of COPD still remains to be discovered. Unbiased approaches, such as genome-wide association (GWA) studies, are critical to identify genes and pathways and to verify suggested genetic variants. Indeed, most of our current understanding about COPD candidate genes originates from GWA studies. Experiments in form of cross-study replications and advanced meta-analyses have propelled the field towards unravelling details about COPD's pathogenesis. Here, we review the discovery of genetic variants in association with COPD phenotypes by discussing the available approaches and current findings. Limitations of current studies are considered and future directions provided.

## Introduction

The Global Initiative for Chronic Obstructive Lung Disease (GOLD) defines chronic obstructive pulmonary disease (COPD) as a disease state associated with airflow obstruction that is not fully reversible (http://www.goldcopd.org/). COPD is currently the fourth leading cause of death and the World Health Organization reports a likely increase in importance to the third leading cause by 2030. According to the World Health Organization, COPD is the most common serious chronic disease worldwide affecting about 64 million people (The global burden of disease: 2004 update, published in 2008). Hence, COPD represents a large and increasing burden to the health care system. Unfortunately, we have limited disease-modifying therapy for COPD and hence, an improved understanding of pathogenetic mechanisms leading to novel therapeutic interventions and preventive strategies is greatly needed. Understanding the genetic predisposition to COPD is essential to develop personalized treatment regimens (Shapiro, 2011). This Review aims to highlight the advances in the discovery of genetic variants in association with COPD by discussing the available approaches and current findings.

**Figure 1:**
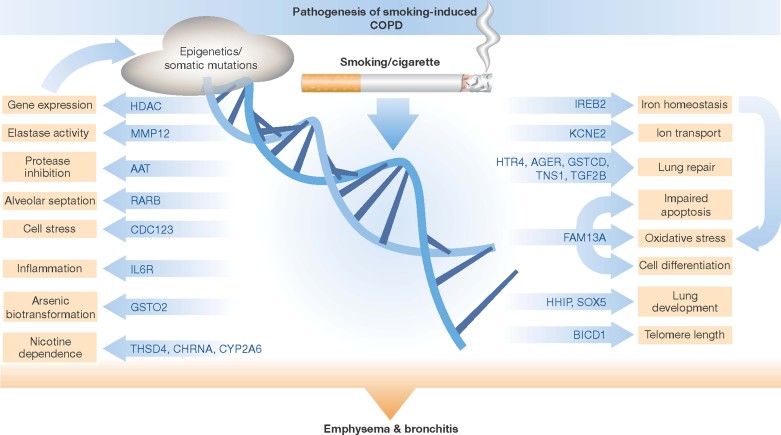
COPD is caused by chronic environmental insults (in particular cigarette smoking) in individuals with predispositions due to variations in one or multiple genes. The combination of environment and genes lead to distinct aberrant pathophysiological processes/pathways, the combination of which causes COPD.

Chronic obstructive pulmonary disease is a multi-factorial disorder caused by environmental determinants – most commonly cigarette smoking – and genetic risk factors (Decramer et al, 2012). In addition to cigarette smoking, COPD can also be caused by other environmental factors, particularly indoor biomass smoke exposure in developing countries (Kennedy & Chambers, 2007). COPD is diagnosed by spirometry showing an irreversible decrease in forced expiratory volume in 1 s (FEV_1_) and the ratio of FEV_1_ to forced vital capacity (FEV_1_/FVC). Although there is a dose–response relationship between FEV_1_ and the amount of smoke exposure, the FEV_1_ decline for smokers with similar exposure varies considerably (Burrows et al, 1977; Fletcher, 1976). This suggests that, in addition to cigarette smoking (and potentially other environmental factors), COPD is also influenced by genetic risk factors (Fig 1). For over 45 years, we have known that genetic variants in the alpha-1 antitrypsin (AAT) gene serpin peptidase inhibitor, clade A, member 1 (*SERPINA1*) lead to COPD. However, AAT deficiency accounts for only 1–2% of all COPD cases. Thus, other variants in the genome are likely to be associated with COPD traits. Finally, it will be important to unravel how environment and genes interact as part of COPD's pathogenesis. As with other chronic inflammatory diseases, it has been shown that epigenetic changes (Yao & Rahman, 2012) and somatic mutations (Tzortzaki et al, 2012) are involved in the pathogenesis of COPD.

Like many chronic complex diseases, it has been difficult to unravel the genetic predisposition and pathogenetic mechanisms for COPD. This is in part due to the heterogeneous nature of the disease. For example, airflow obstruction that defines COPD can result from destruction and enlargement of alveoli (*i.e.* emphysema) with loss of elastic recoil or through obstruction of small airways or both (Hogg et al, 2004). Both of these processes occur with smoking but are not mechanistically related. Therefore, identifying the genetic basis for either of the traits does not justify extrapolation of genetic determinants for other phenotypes. Rather different phenotypic traits may be determined by complex genetic networks, which may or may not overlap. Improved phenotypic measurement of discrete disease traits, such as computerized tomography (CT) for emphysema and spirometry primarily for small airway disease, will allow investigators to more precisely identify genotype–phenotype correlations (Kim et al, 2009).

## Genetic approaches

### Family, twin and segregation studies

Basic genetic approaches included family, twin and segregation studies. Early epidemiological studies found that COPD aggregates in families (Larson et al, 1970; Higgins et al, 1984; Tager and Speizer, 1976) by showing stronger correlations between parents and children or siblings than between spouses. Twin (Redline et al, 1987; Redline, 1990) and segregation studies (Givelber et al, 1998) suggested that the genetic susceptibility for COPD is due to many genes with small effects (Chen et al, 1996; Givelber et al, 1998). These early discoveries initiated the search for novel gene variants with gene-association and linkages studies.

### Candidate gene-association studies

Candidate gene-association studies examine genes that were postulated to play a central role in COPD pathogenesis and investigate the strength of association between disease traits and candidate gene variants. Genetic studies for COPD were performed as gene-association studies by focusing primarily on genes from the protease–antiprotease and oxidant–antioxidant pathways. However, given the diverse pathways (such as inflammation, innate immunity, cell death, matrix repair mechanisms and lung development) involved in COPD pathogenesis it is likely that other genes contribute as well. Also, inconsistencies among those studies restrained our advancement towards clarifying the genetic basics of COPD. The contradictory findings were mostly driven by limited population cohorts, non-standardized disease definitions and varying statistical methods (including differences in adjusting for race, ethnicity, gender, environment and genetic background). A recent meta-analysis of assumed genes showed that many of the gene variants tested in gene-association studies are indeed not successfully associated with COPD (Smolonska et al, 2009). Nevertheless, in spite of the overall disappointing results, a few studies appear promising – namely for MMP12 – and will be discussed in detail below (Hersh et al, 2011; Hunninghake et al, 2009).

**Glossary**

**Candidate gene-association studies**

A candidate gene association study examines the associations between a previously specified gene and the phenotype of interest.

**Chronic obstructive pulmonary disease (COPD)**

A progressive lung disease that makes it hard to breath.

**Computerized tomography (CT)**

Medical imaging procedure that utilizes computer-processed X-rays to produce tomographic images or ‘slices’ of specific areas of the body.

**Family, twin, and segregation studies**

Family and twin studies are association studies that aim to avoid potential confounding factors of population stratifications by using family members for control and cases. Segregation studies determine if a major gene is associated with a phenotype of interest.

**Forced expiratory volume in one second (FEV_1_)**

The volume of air that can forcibly be blown out in 1 s, after full inspiration.

**Genetic variants**

Variations of genomes between members of species or between groups of species. Includes SNP (in case it is a common genetic variant), mutation (in case it is a rare genetic variant) and copy-number variation.

**Genome-wide association (GWA) studies**

Examination of many common genetic variants in different individuals to investigate if any variant is associated with a certain trait.

**Linkage disequilibrium**

The occurrence in a population of two linked alleles at a frequency higher or lower than expected on the basis of the gene frequencies of the individual genes.

**Linkage study**

The formal study of the association between the inheritance of a condition in a family and a particular chromosomal locus.

**Meta-analysis**

Method focused on contrasting and combining results from different studies, in the hope of identifying patterns among study results, sources of disagreement among those results or other interesting relationships that may come to light in the context of multiple studies.

**Next-generation sequencing**

High-throughput sequencing; technology that technologies that parallelizes the sequencing process, producing thousands or millions of sequences at once.

**Pack year**

A way to measure the amount a person has smoked over a long period of time. Calculated by multiplying the number of packs of cigarettes smoked per day by the number of years the person has smoked.

**Pathogenesis**

The mechanism by which the disease is caused.

**Polymorphic marker**

A length of DNA that displays population-based variability so that its inheritance can be followed.

**Single nucleotide polymorphism (SNP)**

DNA sequence variation occurring when a single nucleotide in the genome differs between members of a biological species or paired chromosomes in an individual.

**Spirometry**

Measuring of breath; the most common of pulmonary function tests, measuring lung function, specifically the amount (volume) and/or speed (flow) of air that can be inhaled and exhaled.

**Whole-exome sequencing**

Technique to selectively sequence the coding regions of the genome.

### Linkage studies

As opposed to candidate gene-association studies where genes are chosen, linkage studies represents an unbiased approach and are not limited by an incomplete understanding of disease pathogenesis. Polymorphic markers that are spread across the entire genome are examined for their association with the phenotype of interest. Yet, due to the low marker density, the identified loci are often large in size and can contain several hundreds of genes that need to be sorted through to find those that are associated with the disease. Fine-mapping procedures can eventually narrow the regions to more defined locations and potentially identify novel genes (DeMeo et al, 2006; Wilk et al, 2003). However, linkage studies lack the statistical power needed to identify genetic loci with small genetic effects that are commonly associated with complex diseases, such as COPD (Risch & Merikangas, 1996). Since the recent availability of high-density single nucleotide polymorphism (SNP) panels for whole-genome association studies, linkage studies have largely been abandoned.

### Genome-wide association studies

Genome-wide association (GWA) studies provide an unbiased and hypothesis-free approach to identify genome variations associated with disease phenotypes (Soler Artigas, 2012). We have come a long way since the first COPD GWA study and have not only identified novel candidate genes but also improved the methods along the way to ensure the most accurate results. Due to the use of dense SNP maps (generally hundreds of thousands of SNPs), the search for novel genes can be pinpointed more accurately than with linkage analysis. However, GWAS studies also have limitations due to the small sample sizes (the genome variation underlying lung function are believed to have modest effects; therefore, very large populations are required to identify them) and lack of large-scale follow up studies, which increases the risk for identification of false-positive associations. Also, SNP panels often do not represent disease-associated genetic variants *per se* but may rather be in linkage disequilibrium (LD) with them. A potential strategy to resolve these issues has been proposed recently at an international COPD genetics conference, where it was suggested that a COPD Genetics Consortium be formed to promote collaborations between investigators of existing COPD populations (Silverman et al, 2011). A similar approach has been initiated with the SpiraMeta Consortium combining multiple GWA studies on subjects with European ancestry in large-scale meta-analysis (Obeidat et al, 2011). These Consortia provide an approach for empowering GWA studies and accelerating the identification of common genome variations associated with COPD.

In the very near future, we are going to be able to utilize whole-genome information obtained by next-generation sequencing that will not only improve our abilities to identify common variants but also help teasing out the role of rare and structural genomic variations. However, there are many challenges that must be overcome before whole-genome sequencing becomes routine. For Freeman–Sheldon syndrome 2 and Miller syndrome, it has already been demonstrated successfully that whole-exome sequencing can identify the underlying disease gene (Biesecker, 2010; Ng et al, 2010). Whole-exome sequencing was also applied successfully for the identification of *DNMT3A* mutations in acute myeloid leukaemia (Ley et al, 2010). While whole-exome sequencing has the advantage of cost and coverage, rapid cost reductions of whole-genome sequencing will likely render whole-exome sequencing less useful since it only covers 1–2% of the genome – albeit an important 1–2%.

In summary, although progress in resolving the genetic basis of COPD has been slow since the discovery of AAT in the early 1960's, recent techniques have greatly improved and advances in defining COPD genes have accelerated and will continue to do so. To date, there are currently accepted and recently suggested COPD genes that will be discussed in this review below (Table 1 and Fig 1).

**Table 1:** Overview of COPD genes and details of their study of origin

Year	Gene	Chr	Band	Approach	Phenotype	#SNPs	Population	Primary population(s)	Replication population	Potential Function of Variants	Reference
1964	*AAT*	14	q32.13	Pi system (electrophoresis)	Respiratory insufficiency	NA	2 patients		NA	Protease inhibition	Eriksson (1964)
2007	*IL6R*	1	q21.3	GWA study	FEF25–75	70,987	1220 (fb)	FHS	NA	Immune mechanisms	Wilk et al (2007)
2007	*GSTO2*	10	q25.1		FEV1, FVC				NA	Arsenic biotransformation	
2009	*HHIP*	4	q31.21	GWA study	FEV1/FVC	550,000	7691 (fb)	FHS	Family Heart Study, CHARGE Consortium, SpiroMeta Consortium	Lung development by hedgehog signaing	Wilk et al (2009)
2009	*IREB2*	15	q25.1	GWA study	FEV1/FVC	561,466	823 (810)	Bergen cohort	ICGN, NETT/NAS, BEOCOPD	Pulmonary iron homeostasis	Pillai et al (2009)
2009	*MMP12*	11	q22.3	Gene-association study	FEV1	SNPs in linkage with *MMP12*	8300	Genetics of Asthma in Costa Rica Study, CAMP, Children, Allergy, Milieu, Stockholm, Epidemiological Survey, BEOCOPD, NETT, Lovelace Smokers Cohort, NAS		Elastase activity	Hunninghake et al (2009)
2010	*FAM13A*	4	q22.1	GWA study	FEV1/FVC	550,000	2940 (1380)	Bergen cohort, NETT/NAS, ECLIPSE	COPDGene, ICGN, BEOCOPD, CHARGE Consortium	Oxidative stress and impaired apoptosis	Cho et al (2010)
2010	*GSTCD*	4	q24	GWA study	FEV1	2,705,257	20,288	12 GWA studies (european origin)	CHARGE Consortium	Developmental and remodeling pathways	Repapi et al (2010)
	*TNS1*	2	q35		FEV1						
	*AGER*	6	p21.3		FEV1/FVC				CHARGE Consortium		
	*HTR4*	5	q32		FEV1				CHARGE Consortium		
	*THSD4*	15	q23		FEV1/FVC						
2011	*BICD1*	12	p11.21	GWA study	CT scan	550,000	2380	ECLIPSE, NETT/NAS, Bergen cohort		Telomere shortening	Kong et al (2011)
2011	*SOX5*	12	p12.1	GWA study/Gene-association study	FEV1, FEV1/FVC	1387	386 (424)	NETT/NAS	BEOCOPD	Development lung morphogenesis	Hersh et al (2011)
2011	*MFAP2*	1		Meta-analysis GWA	FEV1, FEV1/FVC	∼2,500,000	48,201	SpiroMeta Consortium, CHARGE Consortium	CARDIA, CROATIA-Split, LifeLines, LBC1936, MESA-Lung, RS-III, TwinsUK-II	antigen of elastin-associated microfibrils	Soler Artigas et al (2011)
	*TGFB2*	1								Epithelial repair process, extracellular collagen accumulation	
	*HDAC4*	2								regulation of gene expression	
	*RARB*	3								premature alveolar septation	
	*MECOM*	3									
	*SPATA9*	5									
	*ARMC2*	6									
	*NCR3*	6									
	*ZKSCAN3*	6									
	*CDC123*	10								Response to cell stress	
	*C10orf11*	10									
	*LRP1*	12									
	*CCDC38*	12									
	*MMP15*	16									
	*CFDP1*	16									
	*KCNE2*	21								Ion transport in airway epithelial cells	
2011	*RAB4B*	19	19q13	Meta-analysis GWA	COPD, FEV1	∼6,100,000	3499 (1922)	ECLIPSE, NETT/NAS, Bergen cohort, COPDGene	ICGN		Cho et al (2012)
	*EGLN2*										
	*MIA*										
	*CYP2A6*									Nicotine dependence	

## Accepted COPD genes

Alpha-1 antitrypsin, encoded by the SERPINA1 gene, is a member of the serpine protease inhibitor superfamily (SERPIN). AAT is mainly produced in the liver and is the major physiologic inhibitor of the serine protease neutrophil elastase (NE; Stoller & Aboussouan, 2011). In addition to NE, AAT inhibits other serine proteinases including proteinase 3 (PR3) (Esnault et al, 1993) and cathepsin G (Topic et al, 2009). AAT also inhibits kallikreins (Felber et al, 2006), matriptase (Janciauskiene et al, 2008), caspase-3 (Miller et al, 2007) and ADAM-17 (Bergin et al, 2010).

Alpha-1 antitrypsin deficiency was first described in 1964 in two patients with severe respiratory insufficiency due to emphysema (Eriksson, 1964). The identification of the AAT variant was possible due to the development of the Pi system, in which AAT mutants migrate distinctly in an electric field from the normal M form. The most common variant, the Z isoform, is due to the single amino acid substitution from glutamic acid to lysine (*i.e.* Glu342Lys), which causes a perturbation in the protein structure resulting in its defective secretion from hepatocytes (Kass et al, 2012). This remarkable story not only shows how a clinical diagnosis can successfully be linked to the genetic basis for a COPD phenotype, it also highlights the long time span required in the past to go from clinical observation (1963) to identification of the amino acid substitution (1978) with limited tools. Fortunately, technical advances in unravelling the pathogenetic basis of diseases greatly accelerate the processes involved in gene finding today. However, at present, the Z variant of AAT remains the only truly accepted genome variant associated with COPD.

## Suggested COPD genes

### Early COPD GWA studies: interleukin 6 receptor (IL6R) and glutathione S-transferase (GSTO2)

Wilk and colleagues reported a GWA study for lung function measures in 2007 (Wilk et al, 2007). The authors collected several spirometry parameters from 1220 related individuals that participated in the Framingham Heart Study (FHS) and performed association studies using 70,987 SNPs from the Affymetrix 100K SNP GeneChip. The location of the strongest associations differed depending on the physiological phenotype. Percent predicted forced expiratory flow from the 25th to 75th percentile (FEF_25–75_) was slightly associated with a SNP in the IL6R region on 1q21 (rs4129267; *p*-value = 0.07), whereas FEV_1_ and FVC were most significantly associated with the GSTO2 region on 10q25.1 (rs156697; *p*-value = 9.42 × 10^−5^). Although the findings of this study were groundbreaking at the time, there were shortcomings. In particular, it is important to notice that both associations did not reach genome-wide significance. While the non-synonymous SNP of GSTO2 reached a *p*-value of 10^−5^, the SNP at the *IL6R* locus only reached a *p*-value of 0.07. Most likely, these shortcomings were at least partially due to the low-density genome coverage with <100,000 SNPs, which may have given rise to potentially ill-defined associations.

IL6R, the receptor of interleukin 6 (IL6), is involved in both pro- and anti-inflammatory processes. IL6R exists as soluble form and forms a complex with IL6. The IL6/IL6R complex appears to play a role in cigarette smoke-induced inflammation, recruiting inflammatory cells to the lung to eliminate foreign particles such as cigarette smoke components, only to have a myriad of other effects on lung tissue. Finally, IL6 (the IL6R ligand) has been shown to be associated with lung function in the Framingham offspring population (Walter et al, 2008).

GSTO2, a family member of the glutathione S-transferases, which are proteins involved in metabolizing xenobiotics and carcinogens, has been postulated to play role in COPD related to its involvement in arsenic biotransformation as arsenic is a chemical element of cigarette smoke (Mukherjee et al, 2006).

### Hedgehog-interacting protein (HHIP)

Two years after the first COPD GWA publication, the investigators published again on findings from the FHS population addressing some of the issues discussed in their initial study (Wilk et al, 2009). Foremost, the SNP panel was more than five-times larger with 550,000 SNPs. Also, the number of subjects was increased from 1220 to 7691. Another advantage of this investigation was that significant SNPs were also tested in a second unrelated population – the Family Heart Study cohort. This time, the investigators examined FEV_1_/FVC to characterize patients. Four linked SNPs on chromosome (Chr) 4 at about 145 Mb (*i.e.* 4q31) were identified to be significant on a genome-wide level. One of those four SNPs (rs13147758) was genotyped in the Family Heart Study, but in this replication study, it did not reach genome-wide significance. However, other studies found SNP associations on 4q31 (Hancock et al, 2010; Repapi et al, 2010; Zhou et al, 2012), thus strengthening evidence that this locus harbours a novel COPD gene. The SNPs on Chr 4 were found to be located in an intergenic region just downstream of the 5′ start site of HHIP, hence representing a potential role in the regulation of HHIP expression. Alternatively, these SNPs could also be in linkage with the disease-causing variant. Together, these findings suggest compelling evidence that this candidate locus may truly influence airflow obstruction in COPD patients. HHIP, a hedgehog-interacting protein, is involved in hedgehog signalling and has been shown to be involved in lung development (Shi et al, 2009). The process of lung development is relevant to COPD because abnormal lung development could lead to impaired reserve predisposing to COPD in smokers. Also, it has been shown that other lung growth and remodelling genes such as *WNT* are re-activated (Tzortzaki et al, 2012), which indicates that abnormal remodelling and repair mechanisms are important molecular processes involved in COPD.

### α-Nicotinic acetylcholine receptor (CHRNA 3/5) locus and iron-responsive element binding protein (IREB2)

At the same time the HHIP candidate locus was published, Pillai et al published a GWA study on the identification of the CHRNA 3/5 locus at 15q25.1 (Pillai et al, 2009). Here, the primary study population was a case-control cohort from Bergen, Norway, with 823 COPD patients and 810 control subjects. The top 100 associations were further investigated in three other cohorts: the International COPD Genetics Network (ICGN; cases and controls), the US National Emphysema Treatment Trail (NETT; COPD cases) and the Normative Aging Study (NAS; controls), as well as the Boston Early-Onset COPD (BEOCOPD) cohort. Similar to the HHIP publication, the phenotypes investigated here were FEV_1_/FVC and post-bronchodilator FEV_1_ (only in the BEOCOPD). Two SNPs on Chr 15 at the CHRNA 3/5 locus (rs8034191 and rs1051730) reached genome-wide significance and were replicated successfully in the independent study cohorts. This Chr 15 locus was previously studied in association with nicotine dependence and, thus represented a promising candidate region (Berrettini, 2008; Saccone et al, 2007; Siedlinski et al, 2011). Interestingly, the SNP associations were significant with and without adjustment for smoking exposure in the original Norway cohort and a significant SNP by pack-years interaction was observed in the ICGN replication population. These observations inferred that the differences between COPD patients and controls were more likely due to genetic determinants of smoking behaviour (*i.e.* nicotine addiction) rather than genetic determinants of COPD *per se*. The latter is supported in light of the observations of significant associations between the CHRNA 3/5 locus and smoking behaviour in lung cancer (Spitz et al, 2008; Thorgeirsson et al, 2008). However, another study on lung cancer did not show that this locus is associated with smoking behaviour (Cantrell et al, 2008). Therefore, further investigation is required to characterize the effects of the Chr 15 locus in regards to smoking behaviour, lung cancer or both. An integrative genomics approach (*i.e.* combined gene expression and genetic association studies) independently identified variants in IREB2 that are in tight LD with the CHRNA 3/5 variants, suggesting IREB2 as a likely COPD candidate gene at the CHRNA 3/5 locus (DeMeo et al, 2009). IREB2 belongs to the iron regulatory protein family (IRPs) that maintains iron homeostasis by regulating iron uptake and distribution. IREB1 and IREB2 maintain the cellular iron metabolism (Rouault, 2006). Regional differences in iron and IRPs exist in smokers (Nelson et al, 1996), which can potentially lead to variation in oxidative stress in the lung – a mechanism of importance in emphysema and lung cancer.

### Family with sequence similarity 13, member A1 (FAM13A)

The independent populations, in which the CHRNA3-CHRNA5-IREB2 and HHIP loci were identified, were combined and resulted in the identification of the FAM13A locus (Cho et al, 2010). Together, the investigators used 2940 COPD cases and 1380 controls (*i.e.* current and former smokers) from three populations: (i) the case–control population from Norway; (ii) a cohort consisting of NETT cases and NAS controls; and (iii) a case and control population from the multi-centre Evaluation of COPD Longitudinally to Identify Predicted Surrogate Endpoints (ECLIPSE). The two most significantly associated SNPs (rs7671167 and rs1903003; *r*^2^ = 0.85) were found at 4q22.1 within a FAM13A intron, which is located just downstream of the Rho-GTPase-activating protein (Rho-GAP) domain. To verify their findings, the investigators genotyped the most significant SNPs using the COPDGene Study population. SNP associations for the top two SNPs were also tested in the ICGN and BEOCOPD populations. Associations of the SNP rs7671167 were significant in COPDGene and ICGN and had a tendency toward significance in the BEOCOPD. Furthermore, an independent GWA investigation of lung function using the populations form the Cohorts for Heart and Aging Research in Genomic Epidemiology (CHARGE) consortium reported an association of FAM13A with FEV_1_/FVC (Hancock et al, 2010). Evidence for a possible role of FAM13A in COPD is its differential expression during hypoxia in cell cultures of epithelial and endothelial cells (Chi et al, 2006) and during epithelial cell differentiation of alveolar type II cells (Wade et al, 2006). FAM13A expression differences have also been observed among mild and severe cystic fibrosis patients (Wright et al, 2006). The significant SNP associations were not associated with pack-years of cigarette smoking and, thus, FAM13A is most likely mediating the genetics of lung function or potentially COPD as opposed to smoking behaviour. A recent report also shows the independent association of the FAM13A locus with lung cancer (Young & Hopkins, 2011).

FAM13A – a Rho-GAP domain containing gene (Cohen et al, 2004) – exhibits tumour suppressor activity by inhibiting the signal transduction molecule Rho A (Ridley, 2001). In COPD Rho A activity has been shown to be involved in oxidative stress and impaired clearance of apoptotic cells (Richens et al, 2009). Similar to HMGCoA reductase inhibitors (statins), Rho-GAP seems to modulate the HMGCoA reductase enzyme, and therefore, provides an explanation why statins may have the potential to protect against COPD and lung cancer (Young et al, 2009).

### Five additional loci associated with FEV_1_ and FEV_1_/FVC

A meta-analysis of several GWA studies by the SpiraMeta Consortium identified five additional loci associated with FEV_1_ and FEV_1_/FVC (Repapi et al, 2010): Tensin 1 (TNS1); glutathione S-transferase, C-terminal domain containing (GSTCD); advanced glycosylation end product-specific receptor (AGER); 5-hydroxytryptamine (serotonin) receptor 4 (HTR4); and thrombospondin, type I, domain containing 4 (THSD4).

As a result of combining multiple GWA studies, the investigators were able to include 20,288 individuals with European ancestry and 54,276 individuals in follow-up investigations. The power of the analysis was greatly increased due to increased quantity of genotype and phenotype data, which ultimately led to the identification of highly significant SNP association (*p*-values ranged from 10^−9^ to 10^−23^). Significant loci were detected for FEV_1_ at 4q24 (GSTCD), 2q35 (TNS1) and 5q33 (HTR4), and for FEV_1_/FVC at 6p21 (AGER) and 15q23 (THSD4). Another locus at 6p21 within the borders of dishevelled associated activator of morphogenesis 2 (DAAM2) contained a suggestive association with FEV1/FVC. GSTCD, HTR4 and AGER were identified independently in the GWA study by the CHARGE Consortium (Hancock et al, 2010). Both, the SpiroMeta and CHARGE Consortia, also found associations at the *HHIP* locus (see above). The associations identified in this study did not change when adjusted for qualitative or quantitative smoking exposure and so the underlying genes most likely are not involved in smoking addiction. Nevertheless, a previous report showed a role for TSHD4 in smoking cessation (Uhl et al, 2008). Proposed mechanisms that may underlie these newly identified genes are either developmental pathways or tissue remodelling pathways that are important for airway architecture and lung repair.

### SRY (sex determining region Y)-box 5 (SOX5)

Linkage studies in the family-based BEOCOPD cohort identified a locus on Chr 12 but the gene of interest could not be isolated at this point (Silverman et al, 2002a, b). Thus, a systematic approach to fine-map the region on Chr 12 was applied by genotyping 1387 SNPs in 386 COPD cases from the NETT cohort and 424 healthy smokers from the NAS cohort (Hersh et al, 2011). Significant associations were located in an intergenic and gene-dense region making the identification of a true candidate gene difficult. Significant SNPs were tried to replicate in the BEOCOPD and ICGN cohorts. The most significant SNP in the BEOCOPD population (rs11046966) was found to be located in close proximity (7 kb downstream) to the 3′ end of SOX5. Further evidence for *SOX5* to be a COPD candidate gene are as follows. COPD subjects showed reduced SOX5 gene expression and abnormal embryonic lung development as well as decreased expression of the extracellular matrix molecule fibronectin were found in Sox5^−/−^ mice. Even though the replication of the SNP was not convincing in one of the replication populations (ICGN), the analysis in the mouse model suggests a role of SOX5 in developmental lung morphogenesis, which, as discussed, could decrease lung functional reserve in the adult.

### Bicaudal D homolog 1 (BICD1)

The investigations that led to the identification of BICD1 were the first to use chest CT scans allowing for specific characterization of the emphysma phenotype (Kong et al, 2011). Up to this point, COPD patients were characterized using spirometry, which is a measure of airflow and not directly related to a single COPD phenotype. Chest CT scans assess lung density, which is proportional to lung airspace enlargement that defines emphysema. Quantitative analysis and radiologist-based qualitative score of CT images were investigated in this GWA study using three different COPD cohorts (*i.e.* ECLIPSE, NETT/NAS, Bergen cohort from Norway). Interestingly, there was only a slight overlap between the quantitative and the qualitative phenotyping methods. The most significant intronic variation on 12p11.21 (rs10844154) was associated with the qualitative assessment by the radiologist but not with the quantitative method. This variation is located close to exon 2 of BICD1. BICD1, a homolog of the Drosophila gene bicaudal-D (BicD), is involved in regulation of dynein function. Exon 2 harbours the binding region for dynein, a molecule involved in mitosis, mRNA transport and dentritic and axonal vesicle transport (Baens & Marynen, 1997). Previously, BICD1 had also been linked to shortening of telomere length (Mangino et al, 2008), supporting recent theories that link COPD to aging (Shapiro, 2011). Telomere shortening triggers cellular senescence, especially in epithelial stem cells. Hence, short telomeres can lead to inability to maintain epithelial integrity leading to emphysema (Alder et al, 2011).

### Sixteen novel genome loci for lung functions

A large-scale meta-analysis in combination with follow-up investigations identified 16 novel genome loci for lung functions (Soler Artigas et al, 2011): Microfibrillar-associated protein 2 (*MFAP2*), Transforming growth factor, beta 2 (*TGFB2-LYPLAL1*), Histone deacatylase 4 (*HDAC4FLJ43879*), Retinoic acid receptor (*RARB*), Ecotropic virus integration site 1 [*MECOM* (*EVI1*)], Spermatogenesis associated 9 (*SPATA9-RHOBTB3*), Armadillo repeat containing 2 (*ARMC2*), Natureal cytotoxicity triggering receptor 3 (*NCR3-AIF1*), Zinc finger with KRAB and SCAN domains 3 (*ZKSCAN3*), Cell division cycle 123 homolog (*CDC123*), Chromosome 100 open reading frame 11 (*C10orf11*), Low density lipoprotein receptor-related protein 1 (*LRP1*), Coiled-coil domain containing 38 (*CCDC38*), Matrix metallopeptidase 15 (*MMP15*), Craniofacial development protein 1 (*CFDP1*) and Potassium voltage-gated channel subfamily E member 2 [*KCNE2-LINC00310* (*C21orf82*)].

The authors evaluated 2.5 million SNPs from 23 individual investigations (17 from the SpiroMeta consortium and 6 from the CHARGE consortium) for FEV_1_ and FEV_1_/FVC in 48,201 individuals of European origin. The association testing, which was stratified for smoking status (ever vs. never smoking), revealed 29 new loci that were associated with lung function at a *p*-value of less than 3 × 10^−6^. Those loci were followed-up in another 17 studies using *in silico* and newly genotyped data. A second meta-analysis across the original and follow-up studies identified SNP associations with *p*-values of <5 × 10^−8^ in 16 of the 29 new loci. Those 16 SNPs are located within or in close proximation to *MFAP2* and *TGFB2-LYPLAL1* on Chr1; *HDAC4FLJ43879* on Chr2; *RARB* and *MECOM* (*EVI1*) on Chr3; *SPATA9-RHOBTB3* on Chr5; *ARMC2*, *NCR3-AIF1* and *ZKSCAN3* on Chr6; *CDC123* and *C10orf11* on Chr10; *LRP1* and *CCDC38* on Chr12; *MMP15* and *CFDP1* on Chr16; and *KCNE2-LINC00310* (*C21orf82*) on Chr21. Some of these new loci are known to be involved in molecular mechanisms that regulate lung functions. For example, *MFAP2* is an antigen of elastin-associated microfibrils (Gibson et al, 1986) and *RARB* has previously been linked to premature alveolar septation (Massaro et al, 2000). *CDC123* plays an important role in response to cell stress by regulation of eukaryotic initiation factor 2 (Bieganowski et al, 2004). *HDAC* has already been recognized in COPD for its regulatory function in gene expression (Ito et al, 2005) and *TGFB2* is known to modulate the epithelial repair processes and extracellular collagen accumulation (Thompson et al, 2006). Finally, KCNE2 is potentially involved in ion transport of airway epithelial cells (Cowley & Linsdell, 2002).

#### Member of RAS oncogen family (RAB4B), Egl nine homolog 2 (EGLN2), melanoma inhibitory activity (MIA), cytochrome P450 2A6 (CYP2A6)

Another large-size meta-analysis GWA study was performed for traits such as COPD, pre-bronchodialator FEV_1_ and severe COPD diagnosed in 3499 cases compared to 1922 controls (Cho et al, 2012). The subjects were obtained from the following four populations: ECLIPSE, NAS and NETT, the Bergen (Norway) cohort and the COPDGene study. Illumina plattforms were used for genotyping and missing SNPs were imputed using the 1000 Genomes data. This study identified a new locus on Chr 19q13 (rs7937), which reached genome-wide significance with a *p*-value of 10^−9^. The association of this locus was repeated in 2859 subjects of the family-based ICGN cohort, thus strengthening the already great evidence for this new locus. Genes within this genome region are *RAB4B*, *EGLN2*, *MIA* and *CYP2A6*. While *RAB4B*, *EGLN2* and *MIA* are of potentially interest due to their expression in developing animal and human lung (Groenman et al, 2007; Lin et al, 2008; Otulakowski et al, 2009), *CYP2A6* has previously been associated with lung cancer and has been shown to be involved in nicotine metabolism (Hukkanen et al, 2005; London et al, 1999; Nakajima et al, 1996), in particular of the major nicotine metabolite cotinine (Thorgeirsson et al, 2010).

## Genes identified by gene-association studies

Early gene-association studies for COPD were often conflicting due to a variety of methodological issues (Silverman, 2006), particularly small sample size and lack of replication populations. However, despite candidate bias, if properly done, these types of studies can be powerful. Hunninghake et al (2009) performed an association study, in which the investigators examined the association between MMP12 variants and the lung function phenotype FEV_1_ (Hunninghake et al, 2009). Unlike many previous association studies, this investigation was well-controlled for age, sex, height and exposure to smoke, and used a very large number of patients. More than 8300 subjects were studied with >20,000 FEV_1_ measurements performed in seven study cohorts [(1). Genetics of Asthma in Costa Rica Study; (2) Childhood Asthma Management Program (CAMP); (3) Children, Allergy, Milieu, Stockholm, Epidemiological Survey; (4) BEOCOPD; (5) NETT; (6) Lovelace Smokers Cohort; (7) NAS]. This scenario greatly improved the power to identify true disease variants. Indeed, the minor allele (G) of a SNP (rs2276109) in the MMP12 promoter region at 11q22.3 was significantly associated with FEV_1_ in all seven cohorts and, particularly, with adult smokers and the risk of COPD in adult smokers.

MMP12 was previously suggested to play a central role in COPD due to its elastase activity and the fact that MMP12 null mutant mice were entirely protected from cigarette smoke-induced emphysema (Hautamaki et al, 1997). The identified variant in the MMP12 promoter mediates decreased promoter activity by diminishing AP-1 binding, which leads to decreased MMP12 expression (Wu et al, 2003). As predicted, less *MMP12* expression protected against COPD. Interestingly, this study also suggests that MMP12 is a candidate gene for asthma, particularly in smokers.

## Animal models to dissect COPD sub-phenotypes

### Phenotype analysis

Animal models were fundamental in formulating the elastase/antielastase hypothesis over 45 years ago, which remains the cornerstone of COPD pathogenesis. At that time, Gross et al (1965) instilled papain into experimental animals resulting in airspace enlargement that defines emphysema (Gross et al, 1965). Subsequently, a variety of animal models have been used to further our understanding of COPD. Models include exposure of animals to molecular, chemical and environmental agents that lead to airspace enlargement (Shapiro, 2000). In particular, elastases (Janoff et al, 1977; Kao et al, 1988; Senior et al, 1977), cigarette smoke (Snider et al, 1986; Wright & Churg, 1990), and more recently, inducers of apoptosis (Kasahara et al, 2000) have been most informative. Over- and under-expression of proteins using transgenic, gene-targeted mice and natural mutant mice have been extremely useful in exploring the pathogenesis of COPD (D'Armiento et al, 1992; Shipley et al, 1996). No single animal model recapitulates human COPD in its entirety, but several result in features associated with the disease (Hautamaki et al, 1997). An advantage of studying COPD as compared to many other diseases is that we know what causes it – cigarette smoke exposure. Of note however, mouse lung structure is not identical to the lung structure in humans. For example, mice have few submucosal glands, they have much less airway branching, and do not contain respiratory bronchioles. However, upon exposure to cigarette smoke, mice do develop important changes similar to humans including inflammation with neutrophils, macrophages and T cells followed by airspace enlargement that is easily detectable in many, but not all, strains at 6 months (Hautamaki et al, 1997). With respect to the airways, upon cigarette smoke exposure, mice lose cilia, develop goblet cell hypertrophy, and show submucosal fibrosis. Importantly, all of these changes are dependent on the individual mouse strain. Indeed, phenotypes measured in multiple mouse strains can be used in GWA scans (genetic mapping studies similar to GWA studies in humans) to identify disease-causing genetic variants.

### Murine genome-wide scans

Using mice in GWA studies can help to accelerate the identification of the genetic basis of complex human diseases. Identifying the genetic basis responsible for phenotypic variations in mouse models is most successful when using dense SNP panels and phenotypic measures across several laboratory strains. It has been suggested that successful genome-wide studies in the mouse require at least 30 different strains (Cervino et al, 2007). In recent years, investigators have performed large-scale phenotyping studies for several disease traits across multiple strains. Currently, high-throughput phenotyping efforts are underway to characterize pathological changes in the lung in response to acute and chronic cigarette smoke exposure. The success of genome-wide scans in the mouse depends on the availability and accuracy of genotype information. SNP panels are available through multiple institutions. For example, several million SNPs for close to 100 mouse strains are provided through the HapMap SNP project (http://snp.cshl.org/) and the Center for Genome Dynamics (http://cgd.jax.org/). Those high-density SNP panels obtained complete SNP coverage across the examined strains by imputations. Depending on the imputation algorithm used to predict missing SNP imputation methods vary in their error rates (Wang et al, 2012). An alternative source for non-imputed genotype information is available by whole-genome sequence data available for 18 strains through the Welcome Trust Sanger Institute (Keane et al, 2011; Yalcin et al, 2011).

Finally, the mouse is a good model for applying advanced bioinformatic techniques to verify the correctness of a potential locus. Identified genes can easily be examined for expression differences at the SNP and mRNA level as well as at the protein level (*i.e.* Western blot or immunohistochemistry). Prediction algorithms such as SIFT by the J. Craig Venter Institute (http://sift.jcvi.org/) or PolyPhen2 by the Sunyaev laboratory at Harvard (http://genetics.bwh.harvard.edu/pph2/) can help to identify functionally important non-synonymous SNPs. Finally, verification of newly discovered genes is possible in genetically engineered mice (*e.g.* transgenic and conditional knockout mice). Studying the genetic basis of COPD in mice may help to tease out molecular pathways that are difficult to unravel due to ethical considerations when investigating human cohorts. Confirmation of the importance of MMP-12 in humans based on mouse studies is one example of the potential to translate findings in mice to humans.

## Future directions

Although we have come a long way since the discovery of AAT, much about the genetic basis of COPD remains to be discovered. The driving factor for understanding COPD susceptibility is to identify true genetic variants. This requires advances in the way we perform genome-wide studies with respect to both phenotyping and genotyping. To understand obstructive lung diseases such as COPD our attention is directed towards improved and more discrete phenotyping. Use of electronic health records will also allow investigators to link individual variation in disease manifestations to underlying genetics. Another limiting factor for successful genome-wide studies is the accuracy and density of the genotype information. The aim is to utilize whole-genome DNA and RNA sequence data so that no imputations become necessary and the SNP density is at its maximum. As cost continues to decrease, use of whole-genome technology is becoming practical for patient populations.

Once genes are identified, we must then put them into molecular pathways or networks and identify the role of these pathways in disease pathogenesis. Unbiased approaches are critical to identify genes and pathways not yet considered. However, many of the discovered genes are not well described and teasing out their function and role in COPD is not always straightforward. This problem is manifest in this Review, where it is not yet possible to place the genes in coherent networks that truly inform about the mechanisms of COPD. Once critical pathways are identified, investigators can work on means to inhibit those pathways leading to disease modifying therapy. Understanding the genetics of COPD is also necessary for the development of personalized medicine. We look forward to a day when genetic information is a routine part of patient care informing the physician of one's disease susceptibility, course, potential complications, co-morbidities and treatment. This, and the elimination of cigarette smoking, will ultimately lower the burden of COPD.

## References

[b1] Alder JK, Guo N, Kembou F, Parry EM, Anderson CJ, Gorgy AI, Walsh MF, Sussan T, Biswal S, Mitzner W (2011). Telomere length is a determinant of emphysema susceptibility. Am J Respir Crit Care Med.

[b2] Baens M, Marynen P (1997). A human homologue (BICD1) of the Drosophila bicaudal-D gene. Genomics.

[b3] Bergin DA, Reeves EP, Meleady P, Henry M, McElvaney OJ, Carroll TP, Condron C, Chotirmall SH, Clynes M, O'Neill SJ (2010). Alpha-1 antitrypsin regulates human neutrophil chemotaxis induced by soluble immune complexes and IL-8. J Clin Invest.

[b4] Berrettini W (2008). Nicotine addiction. Am J Psychiatry.

[b5] Bieganowski P, Shilinski K, Tsichlis PN, Brenner C (2004). Cdc123 and checkpoint forkhead associated with RING proteins control the cell cycle by controlling eIF2gamma abundance. J Biol Chem.

[b6] Biesecker LG (2010). Exome sequencing makes medical genomics a reality. Nat Genet.

[b7] Burrows B, Knudson RJ, Cline MG, Lebowitz MD (1977). Quantitative relationships between cigarette smoking and ventilatory function. Am Rev Respir Dis.

[b8] Cantrell J, Hung D, Fahs MC, Shelley D (2008). Purchasing patterns and smoking behaviors after a large tobacco tax increase: a study of Chinese Americans living in New York City. Public Health Rep.

[b9] Cervino AC, Darvasi A, Fallahi M, Mader CC, Tsinoremas NF (2007). An integrated in silico gene mapping strategy in inbred mice. Genetics.

[b10] Chen Y, Horne SL, Rennie DC, Dosman JA (1996). Segregation analysis of two lung function indices in a random sample of young families: the Humboldt Family Study. Genet Epidemiol.

[b11] Chi JT, Wang Z, Nuyten DS, Rodriguez EH, Schaner ME, Salim A, Wang Y, Kristensen GB, Helland A, Borresen-Dale AL (2006). Gene expression programs in response to hypoxia: cell type specificity and prognostic significance in human cancers. PLoS Med.

[b12] Cho MH, Boutaoui N, Klanderman BJ, Sylvia JS, Ziniti JP, Hersh CP, DeMeo DL, Hunninghake GM, Litonjua AA, Sparrow D (2010). Variants in FAM13A are associated with chronic obstructive pulmonary disease. Nat Genet.

[b13] Cho MH, Castaldi PJ, Wan ES, Siedlinski M, Hersh CP, Demeo DL, Himes BE, Sylvia JS, Klanderman BJ, Ziniti JP (2012). A genome-wide association study of COPD identifies a susceptibility locus on chromosome 19q13. Hum Mol Genet.

[b14] Cohen M, Reichenstein M, Everts-van der Wind A, Heon-Lee J, Shani M, Lewin HA, Weller JI, Ron M, Seroussi E (2004). Cloning and characterization of FAM13A1 – a gene near a milk protein QTL on BTA6: evidence for population-wide linkage disequilibrium in Israeli Holsteins. Genomics.

[b15] Cowley EA, Linsdell P (2002). Characterization of basolateral K+ channels underlying anion secretion in the human airway cell line Calu-3. J Physiol.

[b16] D'Armiento J, Dalal SS, Okada Y, Berg RA, Chada K (1992). Collagenase expression in the lungs of transgenic mice causes pulmonary emphysema. Cell.

[b17] Decramer M, Janssens W, Miravitlles M (2012). Chronic obstructive pulmonary disease. Lancet.

[b18] DeMeo DL, Mariani T, Bhattacharya S, Srisuma S, Lange C, Litonjua A, Bueno R, Pillai SG, Lomas DA, Sparrow D (2009). Integration of genomic and genetic approaches implicates IREB2 as a COPD susceptibility gene. Am J Hum Genet.

[b19] DeMeo DL, Mariani TJ, Lange C, Srisuma S, Litonjua AA, Celedon JC, Lake SL, Reilly JJ, Chapman HA, Mecham BH (2006). The SERPINE2 gene is associated with chronic obstructive pulmonary disease. Am J Hum Genet.

[b20] Eriksson S (1964). Pulmonary emphysema and alpha1-antitrypsin deficiency. Acta Med Scand.

[b21] Esnault VL, Testa A, Audrain M, Roge C, Hamidou M, Barrier JH, Sesboue R, Martin JP, Lesavre P (1993). Alpha 1-antitrypsin genetic polymorphism in ANCA-positive systemic vasculitis. Kidney Int.

[b22] Felber LM, Kundig C, Borgono CA, Chagas JR, Tasinato A, Jichlinski P, Gygi CM, Leisinger HJ, Diamandis EP, Deperthes D (2006). Mutant recombinant serpins as highly specific inhibitors of human kallikrein 14. FEBS J.

[b23] Fletcher CM (1976). Letter: natural history of chronic bronchitis. Br Med J.

[b24] Gibson MA, Hughes JL, Fanning JC, Cleary EG (1986). The major antigen of elastin-associated microfibrils is a 31-kDa glycoprotein. J Biol Chem.

[b25] Givelber RJ, Couropmitree NN, Gottlieb DJ, Evans JC, Levy D, Myers RH, O'Connor GT (1998). Segregation analysis of pulmonary function among families in the Framingham Study. Am J Respir Crit Care Med.

[b26] Groenman F, Rutter M, Caniggia I, Tibboel D, Post M (2007). Hypoxia-inducible factors in the first trimester human lung. J Histochem Cytochem.

[b27] Gross P, Pfitzer EA, Tolker E, Babyak MA, Kaschak M (1965). Experimental emphysema: its production with papain in normal and silicotic rats. Arch Environ Health.

[b28] Hancock DB, Eijgelsheim M, Wilk JB, Gharib SA, Loehr LR, Marciante KD, Franceschini N, van Durme YM, Chen TH, Barr RG (2010). Meta-analyses of genome-wide association studies identify multiple loci associated with pulmonary function. Nat Genet.

[b29] Hautamaki RD, Kobayashi DK, Senior RM, Shapiro SD (1997). Requirement for macrophage elastase for cigarette smoke-induced emphysema in mice. Science.

[b30] Hersh CP, Silverman EK, Gascon J, Bhattacharya S, Klanderman BJ, Litonjua AA, Lefebvre V, Sparrow D, Reilly JJ, Anderson WH (2011). SOX5 is a candidate gene for chronic obstructive pulmonary disease susceptibility and is necessary for lung development. Am J Respir Crit Care Med.

[b31] Higgins MW, Keller JB, Landis JR, Beaty TH, Burrows B, Demets D, Diem JE, Higgins IT, Lakatos E, Lebowitz MD (1984). Risk of chronic obstructive pulmonary disease. Collaborative assessment of the validity of the Tecumseh index of risk. Am Rev Respir Dis.

[b32] Hogg JC, Chu F, Utokaparch S, Woods R, Elliott WM, Buzatu L, Cherniack RM, Rogers RM, Sciurba FC, Coxson HO (2004). The nature of small-airway obstruction in chronic obstructive pulmonary disease. N Engl J Med.

[b33] Hukkanen J, Jacob P, Benowitz NL (2005). Metabolism and disposition kinetics of nicotine. Pharmacol Rev.

[b34] Hunninghake GM, Cho MH, Tesfaigzi Y, Soto-Quiros ME, Avila L, Lasky-Su J, Stidley C, Melen E, Soderhall C, Hallberg J (2009). MMP12, lung function, and COPD in high-risk populations. N Engl J Med.

[b35] Ito K, Ito M, Elliott WM, Cosio B, Caramori G, Kon OM, Barczyk A, Hayashi S, Adcock IM, Hogg JC (2005). Decreased histone deacetylase activity in chronic obstructive pulmonary disease. N Engl J Med.

[b36] Janciauskiene S, Nita I, Subramaniyam D, Li Q, Lancaster JR, Matalon S (2008). Alpha1-antitrypsin inhibits the activity of the matriptase catalytic domain in vitro. Am J Respir Cell Mol Biol.

[b37] Janoff A, Sloan B, Weinbaum G, Damiano V, Sandhaus RA, Elias J, Kimbel P (1977). Experimental emphysema induced with purified human neutrophil elastase: tissue localization of the instilled protease. Am Rev Respir Dis.

[b38] Kao RC, Wehner NG, Skubitz KM, Gray BH, Hoidal JR (1988). Proteinase 3. A distinct human polymorphonuclear leukocyte proteinase that produces emphysema in hamsters. J Clin Invest.

[b39] Kasahara Y, Tuder RM, Taraseviciene-Stewart L, Le Cras TD, Abman S, Hirth PK, Waltenberger J, Voelkel NF (2000). Inhibition of VEGF receptors causes lung cell apoptosis and emphysema. J Clin Invest.

[b40] Kass I, Knaupp AS, Bottomley SP, Buckle AM (2012). Conformational properties of the disease-causing Z variant of alpha1-antitrypsin revealed by theory and experiment. Biophys J.

[b41] Keane TM, Goodstadt L, Danecek P, White MA, Wong K, Yalcin B, Heger A, Agam A, Slater G, Goodson M (2011). Mouse genomic variation and its effect on phenotypes and gene regulation. Nature.

[b42] Kennedy SM, Chambers R, Du W, Dimich-Ward H (2007). Environmental and occupational exposures: do they affect chronic obstructive pulmonary disease differently in women and men. Proc Am Thorac Soc.

[b43] Kim WJ, Silverman EK, Hoffman E, Criner GJ, Mosenifar Z, Sciurba FC, Make BJ, Carey V, Estepar RS, Diaz A (2009). CT metrics of airway disease and emphysema in severe COPD. Chest.

[b44] Kong X, Cho MH, Anderson W, Coxson HO, Muller N, Washko G, Hoffman EA, Bakke P, Gulsvik A, Lomas DA (2011). Genome-wide association study identifies BICD1 as a susceptibility gene for emphysema. Am J Respir Crit Care Med.

[b45] Larson RK, Barman ML, Kueppers F, Fudenberg HH (1970). Genetic and environmental determinants of chronic obstructive pulmonary disease. Ann Intern Med.

[b46] Ley TJ, Ding L, Walter MJ, McLellan MD, Lamprecht T, Larson DE, Kandoth C, Payton JE, Baty J, Welch J (2010). DNMT3A mutations in acute myeloid leukemia. N Engl J Med.

[b47] Lin S, Ikegami M, Xu Y, Bosserhoff AK, Malkinson AM, Shannon JM (2008). Misexpression of MIA disrupts lung morphogenesis and causes neonatal death. Dev Biol.

[b48] London SJ, Idle JR, Daly AK, Coetzee GA (1999). Genetic variation of CYP2A6, smoking, and risk of cancer. Lancet.

[b49] Mangino M, Brouilette S, Braund P, Tirmizi N, Vasa-Nicotera M, Thompson JR, Samani NJ (2008). A regulatory SNP of the BICD1 gene contributes to telomere length variation in humans. Hum Mol Genet.

[b50] Massaro GD, Massaro D, Chan WY, Clerch LB, Ghyselinck N, Chambon P, Chandraratna RA (2000). Retinoic acid receptor-beta: an endogenous inhibitor of the perinatal formation of pulmonary alveoli. Physiol Genomics.

[b51] Miller SD, Greene CM, McLean C, Lawless MW, Taggart CC, O'Neill SJ, McElvaney NG (2007). Tauroursodeoxycholic acid inhibits apoptosis induced by Z alpha-1 antitrypsin via inhibition of Bad. Hepatology.

[b52] Mukherjee B, Salavaggione OE, Pelleymounter LL, Moon I, Eckloff BW, Schaid DJ, Wieben ED, Weinshilboum RM (2006). Glutathione S-transferase omega 1 and omega 2 pharmacogenomics. Drug Metab Dispos.

[b53] Nakajima M, Yamamoto T, Nunoya K, Yokoi T, Nagashima K, Inoue K, Funae Y, Shimada N, Kamataki T, Kuroiwa Y (1996). Characterization of CYP2A6 involved in 3′-hydroxylation of cotinine in human liver microsomes. J Pharmacol Exp Ther.

[b54] Nelson ME, O'Brien-Ladner AR, Wesselius LJ (1996). Regional variation in iron and iron-binding proteins within the lungs of smokers. Am J Respir Crit Care Med.

[b55] Ng SB, Buckingham KJ, Lee C, Bigham AW, Tabor HK, Dent KM, Huff CD, Shannon PT, Jabs EW, Nickerson DA (2010). Exome sequencing identifies the cause of a mendelian disorder. Nat Genet.

[b56] Obeidat M, Wain LV, Shrine N, Kalsheker N, Artigas MS, Repapi E, Burton PR, Johnson T, Ramasamy A, Zhao JH (2011). A comprehensive evaluation of potential lung function associated genes in the SpiroMeta general population sample. PLoS One.

[b57] Otulakowski G, Duan W, O'Brodovich H (2009). Global and gene-specific translational regulation in rat lung development. Am J Respir Cell Mol Biol.

[b58] Pillai SG, Ge D, Zhu G, Kong X, Shianna KV, Need AC, Feng S, Hersh CP, Bakke P, Gulsvik A (2009). A genome-wide association study in chronic obstructive pulmonary disease (COPD): identification of two major susceptibility loci. PLoS Genet.

[b59] Redline S, Tishler PV, Lewitter FI, Tager IB, Munoz A, Speizer FE (1987). Assessment of genetic and nongenetic influences on pulmonary function. A twin study. Am Rev Respir Dis.

[b60] Redline S (1990). Genetic and perinatal risk factors for the development of chronic obstructive pulmonary disease.

[b61] Repapi E, Sayers I, Wain LV, Burton PR, Johnson T, Obeidat M, Zhao JH, Ramasamy A, Zhai G, Vitart V (2010). Genome-wide association study identifies five loci associated with lung function. Nat Genet.

[b62] Richens TR, Linderman DJ, Horstmann SA, Lambert C, Xiao YQ, Keith RL, Boe DM, Morimoto K, Bowler RP, Day BJ (2009). Cigarette smoke impairs clearance of apoptotic cells through oxidant-dependent activation of RhoA. Am J Respir Crit Care Med.

[b63] Ridley AJ (2001). Rho family proteins: coordinating cell responses. Trends Cell Biol.

[b64] Risch N, Merikangas K (1996). The future of genetic studies of complex human diseases. Science.

[b65] Rouault TA (2006). The role of iron regulatory proteins in mammalian iron homeostasis and disease. Nat Chem Biol.

[b66] Saccone SF, Hinrichs AL, Saccone NL, Chase GA, Konvicka K, Madden PA, Breslau N, Johnson EO, Hatsukami D, Pomerleau O (2007). Cholinergic nicotinic receptor genes implicated in a nicotine dependence association study targeting 348 candidate genes with 3713 SNPs. Hum Mol Genet.

[b67] Senior RM, Tegner H, Kuhn C, Ohlsson K, Starcher BC, Pierce JA (1977). The induction of pulmonary emphysema with human leukocyte elastase. Am Rev Respir Dis.

[b68] Shapiro SD (2000). Animal models for COPD. Chest.

[b69] Shapiro SD (2011). Merging personalized medicine and biology of aging in chronic obstructive pulmonary disease. Am J Respir Crit Care Med.

[b70] Shi W, Chen F, Cardoso WV (2009). Mechanisms of lung development: contribution to adult lung disease and relevance to chronic obstructive pulmonary disease. Proc Am Thorac Soc.

[b71] Shipley JM, Wesselschmidt RL, Kobayashi DK, Ley TJ, Shapiro SD (1996). Metalloelastase is required for macrophage-mediated proteolysis and matrix invasion in mice. Proc Natl Acad Sci USA.

[b72] Siedlinski M, Cho MH, Bakke P, Gulsvik A, Lomas DA, Anderson W, Kong X, Rennard SI, Beaty TH, Hokanson JE (2011). Genome-wide association study of smoking behaviours in patients with COPD. Thorax.

[b73] Silverman EK (2006). Progress in chronic obstructive pulmonary disease genetics. Proc Am Thorac Soc.

[b74] Silverman EK, Mosley JD, Palmer LJ, Barth M, Senter JM, Brown A, Drazen JM, Kwiatkowski DJ, Chapman HA, Campbell EJ (2002a). Genome-wide linkage analysis of severe, early-onset chronic obstructive pulmonary disease: airflow obstruction and chronic bronchitis phenotypes. Hum Mol Genet.

[b75] Silverman EK, Palmer LJ, Mosley JD, Barth M, Senter JM, Brown A, Drazen JM, Kwiatkowski DJ, Chapman HA, Campbell EJ (2002b). Genomewide linkage analysis of quantitative spirometric phenotypes in severe early-onset chronic obstructive pulmonary disease. Am J Hum Genet.

[b76] Silverman EK, Vestbo J, Agusti A, Anderson W, Bakke PS, Barnes KC, Barr RG, Bleecker ER, Boezen HM, Burkart KM (2011). Opportunities and challenges in the genetics of COPD 2010: an International COPD Genetics Conference report. COPD.

[b77] Smolonska J, Wijmenga C, Postma DS, Boezen HM (2009). Meta-analyses on suspected chronic obstructive pulmonary disease genes: a summary of 20 years' research. Am J Respir Crit Care Med.

[b78] Snider GL, Lucey EC, Stone PJ (1986). Animal models of emphysema. Am Rev Respir Dis.

[b79] Soler Artigas M (2012). Genome-wide association studies in lung disease. Thorax.

[b80] Soler Artigas M, Loth DW, Wain LV, Gharib SA, Obeidat M, Tang W, Zhai G, Zhao JH, Smith AV, Huffman JE (2011). Genome-wide association and large-scale follow up identifies 16 new loci influencing lung function. Nat Genet.

[b81] Spitz MR, Amos CI, Dong Q, Lin J, Wu X (2008). The CHRNA5-A3 region on chromosome 15q24–25.1 is a risk factor both for nicotine dependence and for lung cancer. J Natl Cancer Inst.

[b82] Stoller JK, Aboussouan LS (2012). A review of alpha-1 antitrypsin deficiency. Am J Respir Crit Care Med.

[b83] Tager IB, Speizer FE (1976). Risk estimates for chronic bronchitis in smokers: a study of male-female differences. Am Rev Respir Dis.

[b84] Thompson HG, Mih JD, Krasieva TB, Tromberg BJ, George SC (2006). Epithelial-derived TGF-beta2 modulates basal and wound-healing subepithelial matrix homeostasis. Am J Physiol Lung Cell Mol Physiol.

[b85] Thorgeirsson TE, Geller F, Sulem P, Rafnar T, Wiste A, Magnusson KP, Manolescu A, Thorleifsson G, Stefansson H, Ingason A (2008). A variant associated with nicotine dependence, lung cancer and peripheral arterial disease. Nature.

[b86] Thorgeirsson TE, Gudbjartsson DF, Surakka I, Vink JM, Amin N, Geller F, Sulem P, Rafnar T, Esko T, Walter S (2010). Sequence variants at CHRNB3-CHRNA6 and CYP2A6 affect smoking behavior. Nat Genet.

[b87] Topic A, Juranic Z, Jelic S, Magazinovic IG (2009). Polymorphism of alpha-1-antitrypsin in hematological malignancies. Genet Mol Biol.

[b88] Tzortzaki EG, Dimakou K, Neofytou E, Tsikritsaki K, Samara K, Avgousti M, Amargianitakis V, Gousiou A, Menikou S, Siafakas NM (2012). Oxidative DNA damage and somatic mutations: a link to the molecular pathogenesis of chronic inflammatory airway diseases. Chest.

[b89] Uhl GR, Liu QR, Drgon T, Johnson C, Walther D, Rose JE, David SP, Niaura R, Lerman C (2008). Molecular genetics of successful smoking cessation: convergent genome-wide association study results. Arch Gen Psychiatry.

[b90] Wade KC, Guttentag SH, Gonzales LW, Maschhoff KL, Gonzales J, Kolla V, Singhal S, Ballard PL (2006). Gene induction during differentiation of human pulmonary type II cells in vitro. Am J Respir Cell Mol Biol.

[b91] Walter RE, Wilk JB, Larson MG, Vasan RS, Keaney JF, Lipinska I, O'Connor GT, Benjamin EJ (2008). Systemic inflammation and COPD: the Framingham Heart Study. Chest.

[b92] Wang JR, de Villena FP, Lawson HA, Cheverud JM, Churchill GA, McMillan L (2012). Imputation of single-nucleotide polymorphisms in inbred mice using local phylogeny. Genetics.

[b93] Wilk JB, Chen TH, Gottlieb DJ, Walter RE, Nagle MW, Brandler BJ, Myers RH, Borecki IB, Silverman EK, Weiss ST (2009). A genome-wide association study of pulmonary function measures in the Framingham Heart Study. PLoS Genet.

[b94] Wilk JB, DeStefano AL, Joost O, Myers RH, Cupples LA, Slater K, Atwood LD, Heard-Costa NL, Herbert A, O'Connor GT (2003). Linkage and association with pulmonary function measures on chromosome 6q27 in the Framingham Heart Study. Hum Mol Genet.

[b95] Wilk JB, Walter RE, Laramie JM, Gottlieb DJ, O'Connor GT (2007). Framingham Heart Study genome-wide association: results for pulmonary function measures. BMC Med Genet.

[b96] Wright JL, Churg A (1990). Cigarette smoke causes physiologic and morphologic changes of emphysema in the guinea pig. Am Rev Respir Dis.

[b97] Wright JM, Merlo CA, Reynolds JB, Zeitlin PL, Garcia JG, Guggino WB, Boyle MP (2006). Respiratory epithelial gene expression in patients with mild and severe cystic fibrosis lung disease. Am J Respir Cell Mol Biol.

[b98] Wu L, Tanimoto A, Murata Y, Sasaguri T, Fan J, Sasaguri Y, Watanabe T (2003). Matrix metalloproteinase-12 gene expression in human vascular smooth muscle cells. Genes Cells.

[b99] Yalcin B, Wong K, Agam A, Goodson M, Keane TM, Gan X, Nellaker C, Goodstadt L, Nicod J, Bhomra A (2011). Sequence-based characterization of structural variation in the mouse genome. Nature.

[b100] Yao H, Rahman I (2012). Role of histone deacetylase 2 in epigenetics and cellular senescence: implications in lung inflammaging and COPD. Am J Physiol Lung Cell Mol Physiol.

[b101] Young RP, Hopkins RJ (2011). COPD and lung cancer linked at a molecular genetic level. Chest.

[b102] Young RP, Hopkins RJ, Christmas T, Black PN, Metcalf P, Gamble GD (2009). COPD prevalence is increased in lung cancer, independent of age, sex and smoking history. Eur Respir J.

[b103] Zhou X, Baron RM, Hardin M, Cho MH, Zielinski J, Hawrylkiewicz I, Sliwinski P, Hersh CP, Mancini JD, Lu K (2012). Identification of a chronic obstructive pulmonary disease genetic determinant that regulates HHIP. Hum Mol Genet.

